# Fatal Rhabdomyolysis in a COVID-19 Patient on Rosuvastatin

**DOI:** 10.7759/cureus.11186

**Published:** 2020-10-26

**Authors:** Zafia Anklesaria, Jonathan Frankman, Jonathan Gordin, Jennifer Zhan, Antonio K Liu

**Affiliations:** 1 Pulmonary and Critical Care, California Hospital Medical Center, Los Angeles, USA; 2 Family Medicine, California Hospital Medical Center, Los Angeles, USA; 3 Cardiology, California Hospital Medical Center, Los Angeles, USA; 4 Emergency Medicine, California Hospital Medical Center, Los Angeles, USA; 5 Neurology, Adventist Health White Memorial, Los Angeles, USA

**Keywords:** covid-19, rhabdomyolysis, rosuvastatin

## Abstract

It is well-established by now that COVID-19 can have a wide variety of neuromuscular manifestations, including rhabdomyolysis. Weakness and elevated creatinine kinase (CK) have been documented as the initial presentation of COVID-19. Myopathy from statin use has also been well-established since the introduction of this class of medication, and the common pathologic mechanism of both entities may have been mitochondrial dysfunction. We present here the case of a COVID-19 patient on rosuvastatin who developed rhabdomyolysis with CK above 1,000,000 units/L. The patient did not present with any respiratory difficulty and responded poorly to treatment, resulting in his untimely demise. COVID-19 may have accentuated an otherwise survivable condition by means of extra stress on mitochondrial homeostasis. Understanding the actual mechanism will be important in the development and utilization of medications in the fight against COVID-19.

## Introduction

We are seven months into the COVID-19 pandemic, and the full neuromuscular manifestation has yet to be understood. Rhabdomyolysis from COVID-19 infection has been described [1-4] but is largely non-fatal. Most reported creatinine kinase (CK) levels are only mildly elevated - less than five digits. One report has a maximum CK of 450,000 [5]. Since COVID-19 is a novel pathogen, the basic mechanism of damage to the muscular system has yet to be elucidated, though an examination of the possible mechanisms will be outlined in the discussion. Myopathy is a known side effect of statin class medication [6]. Its mechanism is believed to be associated with mitochondrial dysfunction and calcium signaling disruption [7]. COVID-19 may have enhanced and exaggerated an otherwise non-fatal muscular pathology with statin use.

## Case presentation

A 57-year-old individual with a past medical history of obesity class 2, hypertension, and dyslipidemia presented to the emergency department (ED) with two days of fever and malaise and one day of generalized weakness with myalgia. Prior to symptom onset, the patient was taking care of family members recovering from COVID-19. There was no known history of renal disease. The patient’s home medications included hydralazine 50 mg twice daily, furosemide 40 mg daily, and rosuvastatin 5 mg daily.

In the ED, vital signs were a temperature of 37.7C (Tmax 39.4C), blood pressure of 160/78, heart rate of 98, respiratory rate of 17, and partial pressure of oxygen (pO2) of 94% on room air. The patient did not initially complain of shortness of breath or chest pain. Physical examination revealed an obese person with diaphoresis, discomfort, and generalized muscle pain. There were coarse and diminished breath sounds bilateral, but no crackles or rales. Cardiac and abdominal examinations were normal. Neurologically, the patient was oriented and coherent, and cranial nerves 2-12 were intact. The patient had diffuse myalgias and weakness that was generalized and non-focal. The patient was able to lift extremities against gravity but was unable to stand up. Notable laboratory findings were a positive COVID-19 PCR from a nasopharyngeal swab. His CBC was unremarkable. Sodium was 118 mmol/L (136-145), potassium was 6.7 mmol/L (3.4-5.0), blood urea nitrogen (BUN) was 47 mg/dL (8.4-25.7), creatinine was 7.6 mg/dL (0.7-1.3), and glomerular filtration rate (GFR) was 7 mL/min/1.73m^2^ (>=60). Calcium was 6 mg/dL (8.4-10.2), total bilirubin was 0.4 mg/dL (0.2-1.2), alanine transaminase (ALT) was 751 units/L (0-55), and aspartate aminotransferase (AST) was 3995 units/L 5-34). Creatinine kinase was 923,120 units/L (30 to 200) and troponin-I was 0.09 ng/mL (<=0.29). The patient had an elevated C-reactive protein (CRP) of >32 mg/dL (0.02-0.5) and an elevated procalcitonin of 17.87 ng/mL (<0.09). Lactic acid was elevated at 2.8 mmol/L (0.5-2.2). Urine was amber and cloudy. Urinalysis had a specific gravity of 1.019 and a pH of 6. Despite large blood on the dipstick, only 6-10 red blood cells were seen on microscopy. ABG revealed a pH of 7.35, partial pressure of carbon dioxide (pCO2) of 22, pO2 of 100, bicarbonate (HCO3) of 12.1, and base excess of -11. Initial chest radiography showed bilateral air space opacities without a significant change on repeat evaluations throughout the admission.

The patient progressed to anuria, and urgent hemodialysis (HD) was initiated following HD catheter placement. Various doses of insulin, sodium bicarbonate, and calcium supplement were administered as needed. Clinically, he remained awake, alert, and interactive with no sensation of dyspnea; muscle soreness subsided but generalized weakness persisted. With the initial round of HD, the CK improved to 560,100 units/L, only to rebound to 1,083,744 units/L the next day. As continuous renal replacement therapy (CRRT) is not available, the patient began undergoing twice daily hemodialysis although acidosis, hyperkalemia, hypocalcemia, and elevated CK would rebound after each round. Despite no symptoms of chest pain, the patient’s troponin-I had a gradual rise to 3.34 ng/mL, although the corresponding CK was 730,660 units/L with a CK-MB of 35.7 ng/mL (<7.1) and CK-MB/CK % of nearly zero.

On the fifth hospital day, the patient was noted to be more tachypneic, with concerns for a tiring respiratory effort, and intubation was performed. No pressor was needed prior to intubation. Immediately following intubation, the patient appeared to have ventricular arrhythmias followed by a pulseless electrical activity (PEA) arrest. Potassium was 6.2 mmol/L shortly prior to intubation. Anticipating the possibility of sudden change, 2 grams of calcium chloride were administered during the code. Advanced cardiovascular life support (ACLS) resuscitative efforts were performed; however, despite prolonged efforts (23 minutes), the patient expired.

## Discussion

Although rhabdomyolysis during acute COVID-19 infection has been reported, CK above one million in a patient on rosuvastatin with fatal outcome has not been described. Besides the patient from Boston with CK in the 450,000 range [5], other reported numbers are lower; two patients reported from New York had CK at 2767 and 1859 [2]. The Wuhan cohort observed elevated CK with muscle pain in 10.7% of severe COVID-19 patients [1]. Although rhabdomyolysis is a reported event in patients on statin therapy, including rosuvastatin, this continues to be a very rare event, with an incidence of about 1.1/10,000 person-years [8]. It is generally believed that statin-induced myopathic toxicity is associated with mitochondrial dysfunction, calcium signaling disruption, and pro-apoptotic signaling [7]. Aging and concomitant infection will further predispose to rhabdomyolysis. With regards to COVID-19's pathophysiologic mechanism of rhabdomyolysis, there is only speculation, with no clear findings. It is recognized that COVID-19 can infect muscle cells by targeting angiotensin-converting enzyme 2 (ACE2) and transmembrane protease serine 2 (TMPRSS2), which is hypothesized to cause mitochondrial dysfunction [3]. If COVID-19 and statin repeatedly stress the muscle cells by the same pathway, a more serious outcome is expected. Additionally, there may be other pathways for COVID-19 to attack myocytes that have not yet been documented.

Protease-like furin has been implicated in the replication and processing of COVID-19 [9]. Furin is present in muscle cells and plays an important role in myalgia caused by the Chikungunya virus [10]. Subsequently, furin inhibitors cause the inhibition of the virus [11]. Without a firm identification of the target protein(s) by which COVID-19 enters our cells, one cannot explain why some patients will have a predominantly muscular system manifestation without significant pulmonary involvement; non-respiratory manifestations of COVID-19 have been discussed lately [12].

Therapeutic consideration is twofold. First, from the standpoint of symptomatic treatment, understanding the disease mechanism will lead to more effective medications. For example, treatments that block COVID-19 entry via ACE2 or furin protease may be beneficial. Secondly, vaccine development relies on what protein COVID-19 targets. Identifying this so-called “point of entry” protein structure will be important for effective and safe vaccine development.

There is an increasing number of publications suggesting statins may be associated with a better outcome in COVID-19 infection [13-14]. An anticipated increase in use, however, will be accompanied by the possible side effects of rhabdomyolysis as described in this case.

## Conclusions

Further case reports, observation, and cohort studies will be needed to analyze if COVID-19 exacerbates the development and severity of rhabdomyolysis in patients on statins. A detailed understanding of the disease mechanism will be needed for the development of medications and vaccines that will restore the cellular mitochondrial balance or prevent the entry of COVID-19 into muscle cells or the cells of other organs. Most importantly, clinicians must be wary of potentially serious rhabdomyolysis in the setting of statin use, which is already widespread and may become more prevalent in the treatment of COVID-19.
